# Recent Advances on the Machine Learning Methods in Identifying DNA Replication Origins in Eukaryotic Genomics

**DOI:** 10.3389/fgene.2018.00613

**Published:** 2018-12-10

**Authors:** Fu-Ying Dao, Hao Lv, Fang Wang, Hui Ding

**Affiliations:** Key Laboratory for Neuro-Information of Ministry of Education, School of Life Science and Technology, Center for Informational Biology, University of Electronic Science and Technology of China, Chengdu, China

**Keywords:** eukaryotic DNA replication, origins of replication, machine learning method, DNA structure properties, webserver

## Abstract

The initiate site of DNA replication is called origins of replication (ORI) which is regulated by a set of regulatory proteins and plays important roles in the basic biochemical process during cell growth and division in all living organisms. Therefore, the study of ORIs is essential for understanding the cell-division cycle and gene expression regulation so that scholars can develop a new strategy against genetic diseases by using the knowledge of DNA replication. Thus, the accurate identification of ORIs will provide key clues for DNA replication research and clinical medicine. Although, the conventional experiments could provide accurate results, they are time-consuming and cost ineffective. On the contrary, bioinformatics-based methods can overcome these shortcomings. Especially, with the emergence of DNA sequences in the post-genomic era, it is highly expected to develop high throughput tools to identify ORIs based on sequence information. In this review, we will summarize the current progress in computational prediction of eukaryotic ORIs including the collection of benchmark dataset, the application of machine learning-based techniques, the results obtained by these methods, and the construction of web servers. Finally, we gave the future perspectives on ORIs prediction. The review provided readers with a whole background of ORIs prediction based on machine learning methods, which will be helpful for researchers to study DNA replication in-depth and drug therapy of genetic defect.

## Introduction

DNA replication is the most essential process in all living organisms and is the basis for biological inheritance. Two identical replicas of DNA generated from one original DNA molecule in the process. The onset of genomic DNA synthesis requires precise interactions of specialized initiator proteins with DNA at sites where the replication machinery can be loaded. These sites, defined as origins of replication (ORIs) (Macalpine and Bell, 2005; Necsulea et al., 2009; Sequeira-Mendes et al., 2009), regulate the beginning of DNA replication. Thus, they play key roles in DNA replication process.

**Figure 1:**
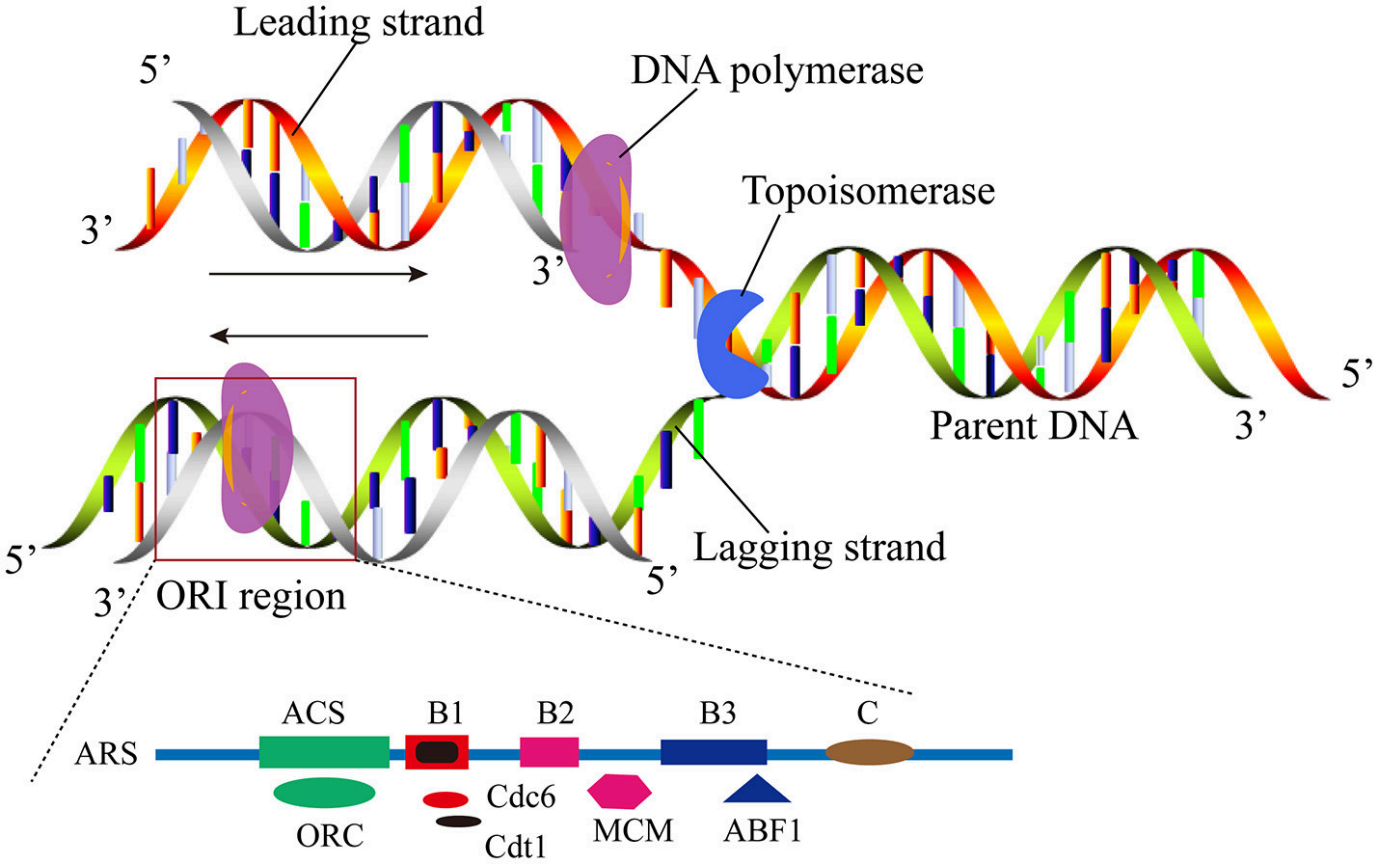
A schematic illustration to show the replication origin in the eukaryote linear DNA molecule. The elements of ORI were found at *S. cerevisiae*, including the ACS and B elements.

It is well-known that the replication mechanisms of prokaryotic and eukaryotic genomes are different. Generally, most of the prokaryotes possess a single circular molecule of DNA with only one ORI (Skarstad and Katayama, 2013). Eukaryotes have more complex DNA replication process than the prokaryotes as shown in Figure 1. One linear chromosome of eukaryotic cell has multiple replicating forks. It has been shown that the number of ORIs is as many as 100,000 in a single human cell (Nasheuer et al., 2002). It ensures DNA replication can be completed in the S phase of the cell cycle timely and speeds the duplication of their much larger store of genetic material. The autonomously replicating sequences (ARS), which contains the specific consensus element autonomous consensus sequences (ACSs) of 11-bp, has been widely distributed in *Saccharomyces cerevisiae* (*S. cerevisiae*) (Stinchcomb et al., 1979; Theis and Newlon, 1997; Dhar et al., 2012). ACS is the binding site for origin recognition complexes (ORC), the main factor that subsequently serves as a landing platform for the assembly of the other pre-RC proteins. Other elements close to the ACS motif contribute to its activity and provide a modular structure to origins (Figure 1) (Marahrens and Stillman, 1992).

Revealing the DNA replication mechanism could provide important clues to understand the regulatory mechanism of cell division and cell cycle. It can also help the discovery of new drugs for the treatment of various diseases (Mcfadden and Roos, 1999; Soldati, 1999; Raghu Ram et al., 2007). Thus, accurate identification of ORIs is an essential prerequisite for further studying and understanding the DNA replication mechanisms. Chromatin immunoprecipitation (Chip) and the next-generation sequencing technology are popular techniques to determine ORIs, which can precisely identify the ORIs (Metzker, 2009; Lubelsky et al., 2012). However, they are expensive and time-consuming for these experimental approaches to perform genome-wide identification of ORIs.

Recent years, with the accumulation of biological experimental data (Levitsky et al., 2005; Yamashita et al., 2011; Gao et al., 2012), it is possible to predict ORIs by computational approaches. Breier et al. (2004) firstly developed an Oriscan algorithm to identify ORIs of *S. cerevisiae*. Shah and Krishnamachari (2012) found the nucleotide correlation measure was better than GC skew to accurately delineate the replication origin. Chen et al. (2012) found that the distribution of DNA bendability and cleavage intensity are different between ORI and non-ORI regions and proposed a support vector machine (SVM) based model to identify ORIs in the *S. cerevisiae* genome. Li et al. (2014) performed a detailed analysis of the compositional bias of *S. cerevisiae* genome. Subsequently, they developed a predictor called iORI-PseKNC (Li et al., 2015) to identify ORIs in *S. cerevisiae* genome. Another web server called iROS-gPseKNC was also established to discriminate ORIs from non-ORIs by using random forest (RF) (Xiao et al., 2016). By combining PseKNC with RF classifier, Zhang et al. (2016) developed a predictor called iOri-Human to identify human ORIs. Recently, Singh et al. (2018) used multi-view ensemble learning (MEL) approach to predict ORIs in *S. cerevisiae* genome. And Liu et al. (2018) developed a new predictor called “iRO-3wPseKNC” to classify four yeast species by rigorous cross-validations.

This review begins with an introduction of benchmark dataset construction for eukaryotic genomes. Then, we outlined machine learning-based techniques that have been applied in ORIs identification successfully and briefly discussed the advantages and limitations of these methods. Next, we analyzed the published prediction results and the published web servers. Finally, future studies on ORI prediction were also discussed.

## Benchmark Dataset

### Published ORI Databases

With the accumulation of biochemical data and the development of computer, and network, more and more databases were constructed to biological data (Huang et al., 2012; He et al., 2016; Feng et al., 2017; Hou et al., 2017; Liang et al., 2017; The Uniprot, 2018). Some have been specially built to store genome replication origin data (Gao and Zhang, 2007; Nieduszynski et al., 2007; Weddington et al., 2008; Cotterill and Kearsey, 2009; Gao et al., 2012; Cherry, 2015). Here, we will briefly introduce these resources.

OriDB is the most extensively used database for identifying eukaryotic DNA replication, in which each potential replication origin site has one of three confidence levels: confirmed, likely and dubious (Nieduszynski et al., 2007). The replication origin information of two organisms budding yeast (*S. cerevisiae*) and fission yeast (*S. pombe*) are stored in the database. Users can access to, search and download ORI data from the database. The database also provides a graphics viewer to allow users to select chromosomal regions and display selected data, which could provide a direct observation and lots of assistance for researchers to study DNA replication.

Another database named DeOri constructed in 2012 which stored eukaryotic ORIs (Gao et al., 2012). A total of 16,145 ORIs were collected from 6 eukaryotic organisms. This database will facilitate the comparative genomic analysis of ORIs, and provide some insight into the nature of ORIs on a genome scale.

In addition to the database described above, there are many other ORI related databases, such as DNAReplication (Cotterill and Kearsey, 2009), Replication Domain (Weddington et al., 2008), and SGD (Cherry, 2015). These databases can be obtained by the URLs in Table 1. And the details of these databases can be referenced the review from Peng et al. (2015).

**Table 1:** A list of published ORI databases.

**Database**	**URL**	**References**
OriDB	http://cerevisiae.oridb.org/	Nieduszynski et al., 2007
DeOri	http://origin.tubic.org/deori/	Gao et al., 2012
DNAReplication	http://www.dnareplication.net/	Cotterill and Kearsey, 2009
ReplicationDomain	https://www2.replicationdomain.com/	Weddington et al., 2008
SGD	https://www.yeastgenome.org/	Cherry, 2015

We found that most of the training datasets of the eukaryotic ORIs recognized researches were structured from database OriDB and only one obtained from DeOri as Table 2 shown. It can be seen that these two databases are reliable and can be used for other studies of ORIs.

**Table 2:** The constructed benchmark data sets for predicting ORIs.

**Datasets**	**ORIs**	**Non-ORIs**	**Total**	**Species**	**Database**	**References**
O1	322	966	1288	*S. cerevisiae*	OriDB	Chen et al., 2012
O2	405	406	811	*S. cerevisiae*	OriDB	Li et al., 2015
O3	283	282	565	*H. sapiens*	OriDB	Zhang et al., 2016
O4	251	410	661	*S. cerevisiae*	OriDB	Singh et al., 2018
		502	753			
		251	502			
O5	340	342	682	*S. cerevisiae*	DeOri	Liu et al., 2018
	338	335	673	*S. pombe*		
	147	147	294	*K. lactis*		
	305	302	607	*P. pastoris*		

### The Published Benchmark Datasets

For the purpose of ORIs prediction, it is necessary to construct an objective and strict benchmark dataset which can be handled by machine learning methods. Based on strict steps (Dao et al., 2017), several previous studies have constructed their own benchmark datasets to train and test their proposed prediction models. The details of these datasets were listed in Table 2.

Based on OriDB, the first benchmark dataset of ORIs called O1 was constructed by Chen et al. (2012). The dataset includes 322 ORIs verified by experiment and 966 non-ORIs in the yeast genome. Li et al. (2015) established the second yeast benchmark dataset named O2, which contains 405 experimentally verified ORIs and 406 non-ORIs. In addition, Zhang et al. (2016) built a new dataset called O3 containing 283 human experimentally confirmed ORIs and 282 human non-ORIs sample on the basis of the DeOri. Singh et al. (2018) gained 251 ARS samples of *S. cerevisiae* from OriDB and generated three negative datasets, respectively. Recently, a dataset (named O5) of four yeast species, including *S. cerevisiae, S. pombe, K. lactis*, and *P. pastoris*, was constructed by Liu et al. (2018).

## ORI Samples Formulation

It is well-known that machine learning algorithms can only handle vectors but not sequence samples (Liu et al., 2016; Yang et al., 2018b). Thus, we should consider how to formulate the ORI sequence with a vector.

### Compositional Analysis Methods

The first method was called GC skew. Since, Lobry (1996) published the computational method to identify ORIs in bacterial genomes in 1996, many scholars have used this method to analyze and identify ORIs (Mclean et al., 1998; Shah and Krishnamachari, 2012; Li et al., 2014; Parikh et al., 2015). For a given ORI sequence, the GC skew can be defined as the following equation.

(1) $$\begin{array}{l} GC\;skew\left[i\right]=\;\frac{f_{i}\left(G\right)-f_{i}\left(C\right)}{f_{i}\left(G\right)+f_{i}\left(C\right)} \end{array}$$

where *f*_*i*_(*G*) and *f*_*i*_(*C*) represent the frequencies of occurrences of Guanine (G) and Cytosine (C) in the *i*-th sliding window along a sequence, respectively. The range of GC skew score is between −1 and +1. Obviously, when *f*_*i*_(*G*) < *f*_*i*_(*C*), the score is a negative value, conversely, it is a positive value. Particularly, the origin of replication is at the position where the GC skew score undergoes an abrupt transition from positive value to negative value.

The GC skew method is the prominent computational measure to predict ORI in the most bacterial genome (Shah and Krishnamachari, 2012). This not only helps to deepen the understanding of advanced biological replication mechanisms, but also contributes to drug discovery. However, this method is not applicable to some bacterial genomes, many archaeal genomes, and almost all eukaryotic genomes (Shah and Krishnamachari, 2012). Moreover, the GC skew is only based on the composition of G and C. Thus, a random sequence displays similar characteristics when it has similar compositions.

The second GC content based method is called GC profile (Li et al., 2014). It is great of importance to acquaint the general compositional features of ORI sequences for understanding the evolution, structure, and function of genomes. For a given ORI sequence, we can obtain the GC profile as Equation (2).

(2) $$\begin{array}{l} GC\;profile\left[i\right]=\frac{f_{i}\left(G\right)+f_{i}\left(C\right)}{f_{i}\left(A\right)+f_{i}\left(C\right)+f_{i}\left(G\right)+f_{i}\left(T\right)} \end{array}$$

where *f*_*i*_(*A*), *f*_*i*_(*C*), *f*_*i*_(*G*), and *f*_*i*_(*T*) represent the frequencies of occurrences of Adenine(A), Cytosine(C), Guanine(G), and Thymine(T) in the *i*-th sliding window along a sequence, respectively. Then, the range of GC profile is between 0 and 1. When the value ranges from 0 to 0.5, the content of GC is lower than that of AT in the windows, conversely, the content of GC is higher than AT content.

GC profile can intuitively give the relationships between the GC content and AT content. A quantitative and qualitative view of genome organization can be easily gained by GC profile. A published tool for studying GC profile can freely available from http://origin.tubic.org/GC-Profile/, which was established by Gao and Zhang (2006). They have provided great convenience for visualizing and analyzing the variation of GC content in genomic sequences.

### Correlation Measure

Two kinds of correlation measures were proposed using ORI prediction. One is the auto-correlation measure which can be defined as:

(3) $$\begin{array}{l} C_{G}=\;\frac{1}{N-1}\sum_{k=1}^{N-1}|\;C\left(k\right)| \end{array}$$

where

(4) $$\begin{array}{l} C\left(k\right)=\;\frac{1}{N-k}\sum_{j=1}^{N-k}a_{j}a_{j+k} \end{array}$$

where *C*(*k*) is the auto-correlation function for a discrete ORI sequence, which was defined in Beauchamp and Yuen (1979) and Cavicchi (2000). There into, *a*_*j*_ ∈ {+1, −1} and the range of the value *j* is between 1 and N. The auto-correlation measure, *C*_*G*_, is the average of all correlation values. The subscript “G” refers to “genome.” The value *C*_*G*_ ranges from 0 to 1. Lower value of *C*_*G*_ indicates lower correlation strength in that one ORI sequence and *vice versa*. For a given nucleic acid sequence ATGTCA, it can be converted into a discrete sequence of bits. When the value of A base is +1, the other three positions (G, C, T) are all −1 and that is similar for each position. Therefore, the sequence can be given rise to four different discrete sequences {1,−1,−1,−1,−1,1}, {−1,−1,1, −1,−1,−1}, {−1,−1,−1,−1,1,−1}, and {−1,1,−1,1,−1,−1} corresponding to the four bases A, T, G, C, respectively. Thus, there are four different bit strings and four different values of correlation strength corresponding to each of the four bases. The detailed usage of the method can be referred to references (Shah and Krishnamachari, 2012; Parikh et al., 2015)

The abrupt change of *C*(*k*) near ORI is helpful to identify ORIs. This method could take into account the order of the bases. However, it did not define the characteristic signature very well. Thus, the cross-correlation measure was developed to identify ORI. It is defined as:

(5) $$\begin{array}{l} C_{CG}=\;\frac{1}{N-1}\sum_{k=1}^{N-1}|\;C_{C}\left(k\right)| \end{array}$$

where

(6) $$\begin{array}{l} C_{c}\left(k\right)=\;\frac{1}{\left(N-k\right)\sigma_{a}\sigma_{b}}\sum_{j=1}^{N-k}\left(a_{j}-\mu_{a}\right)\left(b_{j+k}-\mu_{b}\right) \end{array}$$

where the value of *b*_*j*_ is same as that of *a*_*j*_ in above Equation (6), σ_*a*_ = 1 = σ_*b*_ and μ_*a*_ = 1 = μ_*b*_.

Shah and Krishnamachari (2012) calculated the cross-correlations among A, T and G, C, but they found these values did not give anything meaningful. Therefore, the conclusion can be obtained that a calculation of (A − T)/(A + T) is unable to correctly identify the origin of replication.

### DNA Structural Properties

Chen et al. (2012) analyzed DNA bendability and cleavage intensity around ORIs in the *S. cerevisiae* genome. They found that both DNA bendability and cleavage intensity in core replication regions were significantly lower than those in surrounding regions. Therefore, these two structural properties are of crucial importance in identifying ORIs.

The data of DNA bendability for every trinucleotide in genome was obtained by Brukner et al. (1995), which has also been used in promoter prediction (Abeel et al., 2008; Akan and Deloukas, 2008). Suppose, we calculate the bendability of a sequence CTATG, and its value is 0.406 (0.090[CTA] + 0.182[TAT] + 0.134[ATG]). In a similar way, for a given 300 bp sample sequence, six fragments (300/50) were obtained by using window size of 50 bp with the step of 50 bp. For each fragment, the bendability was calculated. As a result, there are six features for each sample.

Cleavage intensity is the capacity that DNA is unwind by hydroxyl radicals. It can be calculated from parameters for a set of tetra-nucleotide patterns in a given DNA sequence. The parameters of tetra-nucleotides were obtained by experiments (Greenbaum et al., 2007). Subsequently, Bishop et al. (2011) predicted cleavage intensity by ORChID2 algorithm (http://dna.bu.edu/orchid/). Thus, the cleavage intensity of a sequence sample can be calculated by the web tool. By using window size of 50 bp with the step of 50 bp, six features for each sample can be obtained as well.

### Pseudo *K*-Tuple Nucleotide Composition

Stimulating from the concept of pseudo amino acid composition (PseAAC) (Shen and Chou, 2008), the pseudo *k*-tuple nucleotide composition (PseKNC) was developed to deal with DNA/RNA sequences (Chen et al., 2014, 2018b).

The PseKNC is used to formulate samples for predicting ORIs. For an arbitrary DNA sequence D with *L* nucleic acid residues formulated as:

(7) $$\begin{array}{l} \text{D}=R_{1}R_{2}\cdots R_{L-1}R_{L} \end{array}$$

where *R*_*i*_ denotes the nucleic acid residue at the *i*-th position in sample sequence, the sequence can be represented by a 4^*k*^+λ dimension vector as follows.

(8) $$\begin{array}{l} \text{D}=\left[d_{1}d_{2}\cdots d_{4^{k}}d_{4^{k}+1}\cdots d_{4^{k}+\lambda -1}d_{4^{k}+\lambda }\right] \end{array}$$

where

(9) $$\begin{array}{l} d_{u}=\{\begin{array}{ll} \frac{f_{u}}{\sum_{i=1}^{4^{k}}f_{i}+\omega \sum_{j=1}^{\lambda }\theta_{j}}, & \left(1\leq u\leq 4^{k}\right)\\ \frac{\omega \theta_{u-4^{k}}}{\sum_{i=1}^{4^{k}}f_{i}+\omega \sum_{j=1}^{\lambda }\theta_{j}},\; & \left(4^{k}+1\leq u\leq 4^{k}+\lambda \right) \end{array} \end{array}$$

where *f*_*i*_ is denoted as the normalized frequency of the *k*-tuple nucleotide composition in a sequence sample. λ reflects the rank of correlation and is a non-negative integer. ω is the weight factor using to adjust the effect of the sequence correlation. θ_*j*_ is the *j*-tier sequence correlation factor for the sequence, and it can be calculated according to Equations (10)–(12).

(10) $$\begin{array}{l} \theta_{j}=\frac{1}{L-j-1}\sum_{i=1}^{L-j-1}\theta \left(R_{i}R_{i+1},R_{i+j}R_{i+j+1}\right),\;\\ \;\left(j=1,2\cdots \;,\lambda ;\lambda <L\right) \end{array}$$

(11) $$\begin{array}{l} \theta \left(R_{i}R_{i+1},R_{i+j}R_{i+j+1}\right)=\frac{1}{\mu }\sum_{v=1}^{\mu }{\left[P_{v}\left(R_{i}R_{i+1}\right)-P_{v}\left(R_{i+j}R_{i+j+1}\right)\right]}^{2\;} \end{array}$$

(12) $$\begin{array}{l} P_{v}\left(R_{i}R_{i+1}\right)=\frac{P_{v}\left(R_{i}R_{i+1}\right)-<P_{v}>}{SD\left(P_{v}\right)} \end{array}$$

where μ is the number of local DNA structural properties in Equation (11). Six types of local structural parameters are more commonly considered, of which three are local translational parameters (shift, slide, and rise) and the other three are local angular parameters (twist, tilt, and roll) (Guo et al., 2014). *P*_*v*_(*R*_*i*_*R*_*i*+1_) is the numerical value of the *v*-th physicochemical property for the dinucleotide at *i*-th position in an ORI or a non-ORI sample. For the consistency of parameters, a standard conversion should be made before using *P*_*v*_(*R*_*i*_*R*_*i*+1_) in Equation (11). Generally, the *Z*-score is used to normalize the parameters defined in Equation (12) (Chou and Shen, 2006), in there, the symbol < > means the average value of dinucleotides, and *SD* denotes the corresponding standard deviation. The website (http://lin-group.cn/pseknc/default.aspx) was used to calculate PseKNC (Chen et al., 2014).

### Three-Window-Based PseKNC

A new method combined PseKNC with GC asymmetry information to represent sequence information, which named three-window-based Pseudo *k*-tuple nucleotide” or “three-window-based PseKNC'. The concrete procedures are as follows. We suppose D denotes a DNA sample, *L* represents the length of the DNA sequence.

The DNA sequence D is divided into three non-overlapping segments called front window D[1, η], middle window D[η+1, ξ], and rear window D[ξ+1, *L*] according to two parameters ε and δ. Thereinto, ε represents the percentage of total nucleobases of D in the front window, while 1–δ represents the percentage of total nucleobases of *D* in the rear window. And η, ξ are defined as below

(13) $$\begin{array}{l} \{\begin{array}{c} \eta =Int^{c}[L\times \varepsilon ]\\ \xi =Int^{c}[L\times \delta ] \end{array}\;,\;(0\;<\;\varepsilon \;<\;\delta \;<\;1.0) \end{array}$$

where *Int*^*c*^ means taking the ceiling integer for the number in the brackets right after it.

If each subfragment is represented by *k*-tuple nucleotide (or *k*-mers) composition, the DNA sequence will contain 3 × 4^*k*^ components as following shown

(14) $$\begin{array}{l} \text{D}={\left[f_{1}^{1}\ldots f_{4^{k}}^{1}f_{4^{k}+1}^{2}\ldots f_{{2\times 4}^{k}}^{2}f_{{2\times 4}^{k}+1}^{2}\ldots f_{{3\times 4}^{k}}^{3}\right]}^{T} \end{array}$$

where *f*^1^, *f*^2^, *f*^3^ denote the normalized frequency values of the corresponding *k*-tuple nucleotides appearing front, middle, and rear window of sample D, respectively. Thus, a sample sequence can be translated into feature vector as

(15) $$\begin{array}{l} \text{D}={\left[\emptyset_{1}\ldots \emptyset_{4^{\text{k}}+\lambda }\emptyset_{4^{\text{k}}+\lambda +1}\ldots \emptyset_{2\times \left(4^{\text{k}}+\lambda \right)}\emptyset_{2\times \left(4^{\text{k}}+\lambda \right)+1}\ldots \emptyset_{3\times \left(4^{\text{k}}+\lambda \right)}\right]}^{\text{T}} \end{array}$$

Next, the calculation method of **∅**_**u**_ is referred to Type-I PseKNC (Chen et al., 2014). Here, we will not elaborate on the specific calculation method. More details about the three-window-based PseKNC feature extraction method can refer to the research of Liu et al. (2018).

## Prediction Algorithms

### Support Vector Machine

Support vector machine (SVM) (Cao et al., 2014) is a supervised machine learning method based on statistical learning theory, which was developed by Cortes and Vapnik (1995). By seeking the minimum structural risk, the generalization ability of SVM can be improved and the risk of experience can be minimized. Good statistical rules can also be achieved on small training sets. Thus, it is one of the most common and effective classifier. Although, the dimension of biological sequence information is generally high, it is not easy to cause over-fitting problem for SVM. Thus, SVM was widely used in bioinformatics (Jensen and Bateman, 2011; Li et al., 2016; Manavalan and Lee, 2017; Manavalan et al., 2017, 2018a,b,c; Song et al., 2018c; Yang et al., 2018a). The detailed descriptions about SVM can be referred to reference (Vapnik and Vladimir, 1997). In order to reduce the programming burden of researchers, the software package LIBSVM (Chang and Lin, 2011) has be developed and can be freely downloaded from https://www.csie.ntu.edu.tw/~cjlin/libsvm/

Singh et al. (2018) used three classification algorithms (KNN, NB, and SVM) to classify ARS sequences based same feature extracting method, where it was found that SVM is the most reliable classifier. Therefore, SVM is suitable machine learning algorithm for identifying ORIs.

### Random Forest Algorithm

The Random Forest (RF) algorithm Ho (1995, 1998) is an ensemble learning method for classification and regression. It is also widely used in bioinformatics researches (Zhao et al., 2014). RF integrates multiple trees through the idea of integrated learning. The basic unit is a decision tree. Each decision tree is a classifier from an intuitive point of view. *N* trees will have *N* classification results. RF integrates all the classified voting results and specifies the category with the most votes as the final output.

The RF algorithm is flexible and practical. It can handle thousands of input variables without variable deletion and generate an internal unbiased estimate of the generalization error. For estimating missing data and maintains accuracy when a large proportion of the data are missing, the algorithm is still effective.

## Commonly-used Evaluation Metrics

Selecting suitable assessment criteria is helpful for correctly and objectively estimating the proposed model's performance (Chou, 2011; Feng et al., 2013a,b; Chen et al., 2018a; Li et al., 2018a,b; Song et al., 2018a,b). Jackknife test can yield a unique result for a given benchmark dataset, thus, it has been widely used to validate predictors' performance (Yang et al., 2016; Chen et al., 2017). The following four parameters, sensitivity (*Sn*), specificity (*Sp*), overall accuracy (*Acc*), and Mathew's correlation coefficient (*MCC*), are always applied and can be defined as

(16) $$\begin{array}{l} Sn=\frac{TP}{TP+FN} \end{array}$$

(17) $$\begin{array}{l} Sp=\frac{TN}{TN+FP} \end{array}$$

(18) $$\begin{array}{l} Acc=\frac{TP+TN}{TP+TN+FP+FN} \end{array}$$

(19) $$\begin{array}{l} MCC=\frac{TP\times TN-FP\times FN}{\sqrt{\left(TP+FN\right)\times \left(TN+FN\right)\times \left(TP+FP\right)\times \left(TN+FP\right)}} \end{array}$$

where *TP, FP, TN*, and *FN*, respectively denote the number of true positives, false positives, true negatives, and false negatives.

The receiver operating characteristic (ROC) curve (Metz, 1989) can measure the predictive capability of constructed models across the entire range of algorithms' decision values. It is a visual curve graph that shows the model behavior of the *Sn* (the ordinate) against the 1-*Sp* (the abscissa). The area under the ROC (auROC) can objectively assess the performance of a proposed method. auROC = 1 means the model is a perfect classifier, auROC = 0.5 means it is a random predictive classifier.

## Published results

### ORIs Characteristics

Many statistical analyses Chen et al. (2012) and Li et al. (2014) on ORIs have been made for deeply understanding the replication initiation mechanism.

The physiochemical properties of oligonucleotides play important role in replication regulation by analyzing DNA bendability and cleavage intensity around ORIs in the *S. cerevisiae* genomes, Chen et al. (2012) found that both DNA bendability and cleavage intensity in core replication regions were significantly lower than those in in both upstream and downstream regions of ORIs. Based on this result, they proposed DNA physiochemical properties based computational model to predict yeast ORIs.

Li et al. (2014) did a lot of analysis on yeast ORIs. Firstly, they analyzed the compositional bias in the *S. cerevisiae* genome by calculating the GC content surrounding ORIs and found GC content was lower than that of genome-wide. Secondly, they found the scores of GC profile and GC skew in the region of ORIs is significantly lower than that in the flanking regions based on the analysis of the GC profile and GC skew. Thus, they deduced that the replication mechanism of *S. cerevisiae* genome is similar to that of bacterial genomes. Thirdly, by calculating the information redundancies, they found that ORIs sequence have a very strong short-range dominance of base correlations. Fourthly, they investigated the distribution of ORIs in the genome and obtained several conclusions: ORIs always appear in the nucleosome-free regions; promoters might share elements with ORIs; most ORIs are not biased to transcription start regions. Finally, they compared the prediction performance of the above-mentioned characteristics on ORIs prediction by using SVM and found the nucleosome occupancy feature can much more accurately predict ORIs than GC skew and *D*_2_.

### ORIs Prediction

Based on the constructed benchmark datasets listed in Table 2, researchers have developed various models for ORIs prediction by using machine learning methods

On the basis of the benchmark dataset O1, Chen et al. (2012) constructed two models which were, respectively based on structure characteristics (DNA bendability and cleavage intensity) and local word contents of *k*-mer (*k* = 3, 4) by using SVM. They obtained the conclusion that DNA bendability and cleavage intensity could be of great help to ORI prediction. Moreover, they also found that DNA structure characteristics could provide novel insights into regulatory mechanisms of DNA replication. In their structural feature-based model, overall accuracy of 85.86% was achieved with the auROC of 0.848.

Based on the benchmark dataset O2, Li et al. (2015) encoded the ORI sequences of *S. cereviesiae* with PseKNC which could reflect the short-range and long-range sequence-order effects of DNA sequence. They incorporated six common local structural properties of 16 dinucleotides into PseKNC, of which three are local translational parameters (shift, slide, and rise) and the other three are local angular parameters (twist, tilt, and roll). As a result, the overall success rate of 83.72% was achieved in the jackknife cross-validation test based on SVM algorithm. Subsequently, a user-friendly web server called iORI-PseKNC was established and could be freely accessible at http://lin-group.cn/server/iOri-PseKNC. They applied the model in yeast genome and found over 8,000 potential ORIs. Later on Xiao et al. (2016), proposed the dinucleotide position-specific propensity information into the general pseudo nucleotide composition for predicting ORIs by using the RF classifier. As a result, the overall success rate reached 98.03%. According to the model, they provided the web server iROS-gPseKNC which could be obtained from http://www.jcibioinfo.cn/iROS-gPseKNC.

Based on the benchmark dataset O3, Zhang et al. (2016) developed a predictor called iOri-Human. They used the same method as Li et al. (2015) to extract features. The RF algorithm was proposed to perform classification. The overall accuracy in identifying human ORIs was over 75% in jackknife cross-validation. Moreover, a user-friendly web server for iOri-Human has been established at http://lin-group.cn/server/iOri-Human.html, by which users can easily get their desired results without the need to go through the complicated mathematics involved.

Based on the benchmark dataset O4, Singh et al. (2018) compared three classification algorithms namely, distance-based *k*-nearest neighbor (KNN), probabilistic distribution based Naive Bayes (NB) classifier and SVM. They found SVM was a better choice to predict ARS with given properties in all genomic contexts by using the Multi-view ensemble learning model.

Based on the benchmark dataset O5, Liu et al. (2018) established a classification model for ORIs in four yeast species named iRO-3wPseKNC. They employed a different mode PseKNC to extrac features by incorporating the GC asymmetry information into the sample formulation and used the RF algorithm as classification algorithms. According to the jackknife cross-validation, for four yeast species (*S. cerevisiae, S. pombe, K. lactis*, and *P. pastoris*), high success prediction rates were obtained, which were 0.730, 0.965, 0.851, and 0.710, respectively. That clearly indicated the proposed their predictor was indeed quite powerful and may become a very useful bioinformatics tool for genome analysis.

Web server is a newly emerging tool in the internet age. It has brought a lot of convenience to the vast majority of biochemical scholars without the need to understand the mathematical details and programming. The difficult mathematics and computational methods can be easily used by means of web servers. Listed in Table 3 are the overviews of the web servers for ORI prediction as described above. As we can see in Table 3, for a given unknown sequence, predictors, iORI-PseKNC, and iOri-Human, can predict a more accurate ORI position by the 300 bp window but homogeneous species. The iRO-3wPseKNC can classify four different species of yeast for a given sequence but predict a whole given sequence with only one result. And the iROS-gPseKNC can't work.

**Table 3:** A list of the published prediction tools for ORI prediction.

**Name**	**Species**	**URL**	**References**	**Work (Yes/No)**	**Prediction window**
iORI-PseKNC	*S. cerevisiae*	http://lin-group.cn/server/iOri-PseKNC	Li et al., 2015	Yes	300 bp
iROS-gPseKNC	*S. cerevisiae*	https://www.jcibioinfo.cn/iROS-gPseKNC	Xiao et al., 2016	No	–
iOri-Human	*H. sapiens*	http://lin-group.cn/server/iOri-Human.html	Zhang et al., 2016	Yes	300 bp
iRO-3wPseKNC	*S. cerevisiae*	http://bioinformatics.hitsz.edu.cn/iRO-3wPseKNC/	Liu et al., 2018	Yes	None
	*S. pombe*				
	*K. lactis*				
	*P. pastoris*				

## Conclusions and Perspectives

DNA molecule can transfer the genetic information from parent to offspring by replication. Thus, DNA replication plays the one of the most important part of life process at the cellular level. It is fundamentally significant for understanding such vitally important biological process to obtain the knowledge of ORIs. Accurate identification of ORIs will provide crucial clues in revealing DNA replication mechanism and discovering new drugs for treatment of various diseases. The computational tools based on machine learning are especially necessary to acquire these predicting outcomes.

Generally, developing a sequence-based predictor needs to consider the following guidelines (Chou, 2011): (i) benchmark dataset construction; (ii) feature extraction and feature optimization; (iii) classification algorithm comparison and selection; (iv) result evaluation and analysis; (v) web server establishment.

We found that none of these abovementioned publications used feature selection methods to improve prediction accuracy. Feature selection is important in pattern recognition for obtaining key features, excluding redundant information, or noise, improving robust, efficiency, and accuracy of models as well as solving dimension disaster. At present, many feature selection techniques have been proposed to optimize a feature set for producing the maximum accuracy and establishing a robust bioinformatics model, for instance, minimal-redundancy-maximal-relevance (mRMR) (Peng et al., 2005), maximum-relevance-maximum-distance (MRMD) (Zou et al., 2016b), (BD) (Su et al., 2018), *F*-score (Lin et al., 2014), and the analysis of variance (ANOVA) (Tang et al., 2018).

minimal-redundancy-maximal-relevance is a kind of filtering feature method proposed by Peng et al. (2005). The core idea of mRMR is to maximize the correlation between features and categorical labels and at same time to minimize the correlation between features and features. It runs fast and can always produce robust models. MRMD is similar to mRMR but can scan the ranking features for a best dimension. It was widely used in bioinformatics recently (Zou et al., 2016a; Wei et al., 2018c). BD-based feature selection technique has strict and objective statistical foundation for extracting the over-represent motifs in sample sequences (Feng et al., 2018; Su et al., 2018; Zhu et al., 2018). Thus, it is also widely applied for sequence analysis (Feng and Luo, 2008; Lai et al., 2017). F-score, a simple feature selection method is usually used to measure the degree of difference between two real number sets (Lin et al., 2014, 2017). This method could achieve the most effective feature selection with strict mathematical definition. The basic idea of ANOVA is to compare the difference between the variance among groups and the variance within the group under different levels of influence, and then to determine differential expressed features (Chen et al., 2016).

In bioinformatics prediction, a key role for obtaining a highly accurate model is to use valid mathematical descriptors to formulate samples. The Type-II PseKNC is a different kind PseKNC which could reflect the correlation effect for different kind of physiochemical properties (Chen et al., 2014). Thus, it is better than Type-I PseKNC for describing ORI samples. However, it has not been used in all the published references for predicting ORI. In the future, we will try to use the Type-II PseKNC method combined with feature selection techniques to build a powerful and robust prediction model for predicting ORIs.

In summary, although a great progress for ORIs prediction has been obtained, further improvements should be made from the following points. Firstly, most of works focused on the ORIs prediction in bacteria, yeast and human genomes. Thus, we should try our best to construct more models for the prediction of ORIs in other species genomes. Secondly, with more and more accumulation of biochemical data, some old benchmark datasets should be updated constantly to acquire much more reliable samples. Thirdly, appropriate feature selection methods should be employed to reduce feature vector dimensions and improve the prediction accuracy. Fourth, try more machine learning methods to build classification models, such as deep learning (Cao et al., 2016, 2017; Long et al., 2017; Shao et al., 2018; Wei et al., 2018a,b; Yu et al., 2018; Zhang et al., 2018).

## Author Contributions

HD conceived and designed the experiments. F-YD, HL, and FW analyzed the data and reviewed the references. F-YD, HL, FW, and HD performed the analysis and wrote the paper. All authors read and approved the final manuscript.

### Conflict of interest statement

The authors declare that the research was conducted in the absence of any commercial or financial relationships that could be construed as a potential conflict of interest.
